# Low tuberculosis case detection: a community and health facility based study of contributory factors in the Nkwanta South district of Ghana

**DOI:** 10.1186/s13104-016-2136-x

**Published:** 2016-06-29

**Authors:** Gregory K. Amenuvegbe, Anto Francis, Binka Fred

**Affiliations:** School of Public Health, University of Health and Allied Sciences, Ho, Ghana; School of Public Health, University of Ghana, Legon, Ghana

**Keywords:** Case detection, Tuberculosis, Nkwanta south district, Community-based volunteers

## Abstract

**Background:**

Tuberculosis (TB) continues to pose a major public health problem globally. In Ghana, the national TB case detection rate is 81 %; however, some districts are not able to meet their case detection targets. This study was therefore carried out in the Nkwanta South district to identify possible factors contributing to low TB case detection.

**Methods:**

A cross sectional descriptive study involving the review of outpatients records for the year 2012 was conducted. Data on cough for 2 weeks or more duration, age, sex, area of residence and sputum smear examination were extracted. A community-based survey involving household contacts of TB patients and community based volunteers was also carried out. Data collected in the community included knowledge of TB status of relatives, level of socialization with TB patients and signs and symptoms of TB disease. Descriptive statistics including cross-tabulations were used to identify possible factors contributing to low TB case detection.

**Results:**

A total of 932 patients out of 3987 reported coughing for 2 weeks or more (23.4 %; 932/3987). Out of that, only 24.6 % (230/932) had sputum smear microscopy done, yielding 57 (24.8 %) positive cases. Five out of the 57 positive cases were found not registered for the initiation of treatment leading to a false primary default rate of 8.8 % per year. Eighty-five percent of the contacts were able to mention persistent cough as a sign/symptom of TB with 80.4 % indicating that TB can be cured. Only 10 % of health facilities provided diagnostic services in the district with only 25 % of staff having had training in TB management.

**Conclusion:**

The study identified some factors (weak record review systems, inadequate diagnostic centres, lack of trained persons and some level of stigma at the community level) that could be contributing to low TB case detection in the Nkwanta South district.

**Electronic supplementary material:**

The online version of this article (doi:10.1186/s13104-016-2136-x) contains supplementary material, which is available to authorized users.

## Background

Human tuberculosis (TB) is an infectious bacterial disease caused by *Mycobacterium tuberculosis*. The infection is transmitted from person to person via droplets from the throat and lungs of people with active respiratory disease [1]. The disease ranks second in terms of mortality from a single infectious agent, after the human immunodeficiency virus. In 2013, there were about nine million new cases worldwide [2]. Of those who become infected with *Mycobacterium tuberculosis*, 10–12 % will progress to tuberculosis disease [3] after a period ranging from weeks to decades. TB can affect any part of the body but most infections occur in the lungs commonly referred to as pulmonary TB. Infection in other parts of the body is called extra-pulmonary TB. The disease is of public health importance as one-third of the world’s population is infected with the bacteria, resulting in about two million deaths each year [4, 5].

Globally, TB case detection rate has stagnated at around 60 % and has still not reached the 70 % target set for 2005 [6] with sub-Sahara Africa having a much lower rate of 52 % [3]. Poor TB case detection leads to increased transmission and high TB prevalence rates, as each active case has the capacity to infect 10–15 people per year [7].

TB is diagnosed mainly by sputum smear microscopy (SSM) [2] in most endemic countries. At the primary health care delivery level however, resources including human, for sputum smear examination for acid fast Bacilli (AFB) is usually not available. Therefore, most cases of pulmonary tuberculosis are diagnosed based on clinical and radiological indicators [8].

In Ghana, the National TB Control Programme (NTP) was established in 1994 [9]. Before then, TB care was in only regional hospitals and the two TEACHING HOSPITALS where Chest Clinics were used to house TB patients. As part of the health sector response to the TB epidemic in Ghana in recent times a focus has been placed on implementing the expanded framework of the DOTS strategy. Its objective is to identify the sources of infection in the community. Treatment of those infectious patients rapidly renders them noninfectious, thereby breaking the chain of transmission [10].

According to the 2011 TB annual report of Ghana, in the year 2010, the proportion of deaths among TB clients was 7.2 % and treatment success was 85.5 % with a defaulter rate of 3.0 % [11]. The Volta region where the Nkwanta South district is located, has seen slight increases in the number of reported cases, from 1390 in 2010 to 1544 in 2011 and 1579 cases in 2012. However, reported cases from Nkwanta south district were on the decline [12] necessitating the current study in the district. In 2010 for instance, out of the 107 expected cases, only 61 (57 %) were actually detected. In 2011, the proportion of cases detected reduced from 57 % in 2010 to 54.5 %. A further decline in the number of cases detected occurred in 2012 when only 52 cases were reported out of the expected 112 (46.4 %). The purpose of the current study therefore was to determine factors contributing to the low TB case detection rate in the district.

## Methods

### Study area

The study was conducted in the Nkwanta South district of the Volta region of Ghana. The district covers an area of about 4530 km^2^ with a population of 111,197 people. The district has been zoned into four sub-districts around its health facilities to facilitate health service delivery. These are Tutukpene, Bonakye, Brewaniase, and Nkwanta sub-districts. The district capital is Nkwanta. The main occupation of the inhabitants is farming of mainly food crops including yam, cassava and maize.

There are two hospitals (one mission and one government owned), two health centres and 33 Community Health Planning and Services (CHPS) compounds. The district has staff strength of three doctors, 32 nurses (all categories), two pharmacists, two pharmacist technicians, four laboratory technicians and four health information officers. The district also has 298 community child growth promoters and 105 community-based volunteers who are working on integrated disease surveillance and response (IDSR) activities including tuberculosis.

### Study design

A community and health facility based cross-sectional descriptive study was carried out in the Nkwanta South district in the Volta region of Ghana. The health facility component involved review of out patient department (OPD) records covering the period January to December 2012 and a face-to-face interview with the health staffs in-charge of the facilities from which TB cases were reported during the year and technical staff working on the TB programme. Data on coughs that had a history of 2 weeks or more duration were extracted from the hospital folders. The community level data collection involved relatives of registered TB patients for the year 2012 and community health volunteers in the district who do follow-ups and contact tracing of TB patients.

### Records review

A representative sample of routine OPD records from January to December 2012 on patients who sought care at the district hospital were reviewed. Data on reported coughs of 2 weeks or more duration were extracted onto a data extraction sheet specifically designed for this study. These records were cross-checked with records in the laboratory registers on patients who had sputum smear examination done. Patients who were eligible for sputum smear examination but were not found in the laboratory registers were noted. Records on those patients who were sputum smear positive were traced to the treatment registers and verified with all those put on treatment during the period under review. Any inconsistencies between the records in the laboratory registers and the treatment registers that might cause initial defaulting of TB patients were also noted.

### Interview with health staff

During the year 2012, TB cases were reported from 22 health facilities in the district. All the health staff in-charge of the 22 health facilities and the technical staff responsible for the TB programme participated in the study. Data on how follow-ups were carried out on patients who tested positive for TB were collected from these officers. Their institutional record review and TB contact tracing records were also checked (Additional file 1).

### Interview of relatives of TB patients and community health volunteers

Tuberculosis patients captured in the treatment registers for the year 2012 were followed-up to their homes using their addresses and telephone numbers where available. Data were collected from their immediate family contacts using a structured questionnaire. This was to establish whether as a high risk group they have been examined for TB infection. Community health volunteers in the district were also interviewed and data collected on the number of follow-ups done in terms of tracing contacts of TB patients and identification of suspected TB cases as part of their surveillance activities in the district.

### Sample size determination and sampling

In the year 2012, 52 cases of TB of all forms were reported in the district. Using the basic assumption that each active TB case can infect 10–15 people in a year, we multiplied the 52 cases for the year by the lower value of 10 and had 520 as a rough estimate of infected close contacts by the reported cases. Also, the total OPD attendance at the district hospital for the year 2012 was 34,844. Using Glen (1992) formula for sample size estimation a sample of 4500 was deemed adequate to detect any difference between the number of possible TB cases reported to the hospital and the number that had sputum smear microscopy done.

A summarized list from the 2012 TB register was produced and a random list generated using Microsoft excel. These persons were serially followed up in the communities and their close contacts interviewed until the predetermined sample was obtained. For the OPD record review, the systematic sampling method was used to select the patient folders from all the clients who received treatment at the facility between January and December 2012, using a sampling interval of seven.

### Ethical issues

The proposal was submitted to the Ethical Review Committee of the Ghana Health Service for review and approval before the start of the study (ID Number: GHS-ERC: 20/04/14). Written informed consent was obtained from all the study participants. Confidentiality of the patients whose contacts were being followed for data collection was not breached in this study as the process forms part of continuum of care for the patient and the community. The patients were contacted through their contact information in their folders at the hospital and the purpose of the study was discussed with them including follow up to their homes and administering questionnaires to their household contacts. The benefits of their relatives reporting for early treatment in health facilities in case of experiencing signs and symptoms of TB was well explained to them.

### Statistical analyses

The data were double entered and validated in Epidata version 3.1 before being exported into STATA 12 for analysis (Stata Corp, Collage Station). Frequency distributions and descriptive statistics such as mean were calculated. Comparisons between groups were made using the Pearson Chi square test for qualitative/categorical variables (proportions), and the Students’t-test for quantitative variables (means). The dependent variable (cough suspected to be TB) was cross tabulated with sputum smear microscopy examination. All statistical tests were two sided and an alpha level of <0.05 was considered a statistically significant result. The 95 % confidence intervals were also used where appropriate.

### Availability of data and materials

The datasets supporting the conclusions of this article are included within the article and its additional files.

## Results

### Hospital records review and related TB case detection factors

A total of 4500 individual records were sampled and reviewed out of the annual total of 34,844 available records for the period January–December 2012. Most of the hospital attendants (70.0 %; 24,391/34,844) were residents of the district with a few (25.0 %; 8711/34,844) coming from surrounding districts and 5.0 % (1742/34,844) from neighbouring Togo. The analysis was based on 3987 records after incomplete records (those without age and sex variables) [513/4500, 11.4 %] were removed from the dataset. The mean age of the patients was 27.9 years (SD 18.94; range 1–95). Majority of the patients (63.86 %, 2546/3987) were females [(CI 1.0–1.5) p < 0001]. A total of 932 patients (23.38 %) presented with cough of 2 weeks or more duration at the OPD; however, sputum smear microscopy was done for only 24.68 % (230/932) of them, with as many as 702 (75.4 %) of the cough cases going back home without further laboratory investigation for *Mycobacterium tuberculosis* infection [p < 0.0001] (Table 1). A total of 57 of the 230 cough cases (24.9 %) tested positive for TB after sputum smear microscopy. Interestingly, not all the 57 cases were registered to be put on TB treatment, thus, giving a false primary defaulting rate of 9.0 % (5/57).

**Table 1:** Characteristics of study participants

Study group	No.	%	p value	95 % CI
*Reviewed OPD records*				
Total OPD attendance	34,844	–		
Sampled records	4500	12.91		
Complete data	3987	88.6		
Incomplete data	513	11.4	p < 0.0001	
Male	1441	36.14		
Female	2546	63.86	p < 0.0001	1.0–1.5
Cough 2 weeks and more	932	23.38 (932/3987)		
Sputum smear microscopy done	230	24.68 (230/932)	p < 0001	
*Household contacts*				
Female	119	47.60		
Male	131	52.40	p = 0.2	
Resident in rural area	230	91.97		
Resident in urban area	20	8.03	p = 0.067	1.04–1.11
No formal education	126	50.40		
Formal education	124	49.60	p > 0.05	
Unemployed	84	33.60		
Formal sector employment	14	5.60		
Informal sector employment	152	60.80	p = 0.014	

### Household contacts and related TB case detection factors

A total of 52 registered TB patients receiving treatment were traced into their homes. In these homes, 250 potential contacts were identified (mean per household = 4; range 1–8). This number consisted of 131 males (52.40 %) with most of them (91.6 %, 229/250) resident in the rural parts of the district. The mean age was 18.6 years (SD 10.3; range 4–80 years). About half (126/250, 50.4 %) of them had no formal education, with most of them working in the informal sector (farming, fishing, fish mongering, artisanship and trading) (Table 1).

The level of knowledge about TB among the household contacts was found to be high with 89.70 % (227/250) of them mentioning cough as a symptom of TB disease. Only 2.15 % of the contacts could not mention any sign or symptom of TB. A significant number 188 (79.32 %) of the contacts were also able to indicate that TB is transmitted from person-to-person with only 49 (20.68 %) indicating that person-to-person transmission is not possible (p < 001). Similarly, 201 (80.40 %) of the people indicated that TB is curable (p < 0.0001) (Table 2).

**Table 2:** Knowledge on tuberculosis and attitudes of household members towards TB patients

Variable	N = 250, No.	%	p value	95 % CI
*Signs and symptoms*				
Cough	227	89.70		
Chest pain	19	8.06		
Do not know	4	2.15	p < 0.001	1.06–1.18
*Mode of transmission*				
Person-to-person	188	79.32	p < 0.001	1.15–1.25
*Treatment*				
Curable	201	80.40	p < 0.0001	1.14–1.24
*Informed about TB status of relative*				
Informed	59	23.60	p = 0.06	1.18–2.0
*Tested for TB*				
Tested	5	2.00		
Not tested	245	88.37	p = 0001	1.0–1.2
*Cost of TB treatment*				
Free	107	42.80	p < 0.001	1.5–1.7
*Means of accessing health care*				
Health insurance	189	75.60	p = 0.03	1.15–1.25
*Stigma against TB patient*				
Will eat with a non-relative TB patient	70	28.00	p = 0.004	1.23–1.35
Will eat with a relative TB patient	175	70.56	p = 0.05	1.67–1.79

Further analysis revealed that only a small proportion, 23.60 % (59/250) of the household contacts received information on the TB status of their relative in the immediate past 2 years and the need for them to get tested. Consequently, only five out of the 250 contacts (2.0 %, p = 0.0001) were tested for TB on the basis of a relative having TB.

### Community based surveillance volunteers and related TB case detection factors

A total of 105 community based surveillance volunteers (CBSVs) were contacted; their mean age was 39.9 years (range 19–65 years; SD 9.9). Most of them (97.1 %, 102/105) were males, with only 2.9 % (3/105) females. Most of the volunteers (97.16 %; 102/105) were resident in the rural parts of the district and engaged in farming (90/105, 85.71 %). Majority (71.40 %; 75/105), of them had basic level education with 6.67 % (7/105) without any formal education. Of the 105 CBSV contacted only 28 (26.67 %) have had structured training in disease surveillance with the majority 77 (73.33 %) yet to receive any structured form of training [p = 0.01] (Table 3). Twenty-eight of the volunteers (26.42 %) were able to mention three correct signs/symptoms of TB, 40 (37.74 %) were able to mention two correct signs/symptoms while 38 (35.85 %) could mention only one correct sign/symptom of TB.

**Table 3:** Knowledge of tuberculosis and activities of community based surveillance volunteers

Variable	N = 105, No.	%	p value	95 % CI
*Training on TB*				
Trained	28	26.67	p < 0.01	0.64–0.82
*Knowledge on signs and symptoms of TB*				
Mentioned 3 correct signs and symptoms	28	26.42		
Mentioned 2 correct signs and symptoms	39	37.74		
Mentioned 1 correct sign and symptom	38	35.85	p = 0.47	1.2–1.9
*Evidence of CBSV register*				
Register available	33	32.04	p < 0.001	0.6–0.8
*Recorded cases in CBVS register*				
Cases recorded	15	35.71		
Cases not recorded	18	64.29	p = 0.5	0.5–0.8
*Identified possible TB case in past year*				
Identified possible TB case	28	25.24	p = 0.001	0.7–0.8
*Referred suspects to health facility*				
Referred	27	96.43	p < 0.002	0.2–0.4
*Received feedback from DHMT*				
Feedback received	10	9.52		
No feedback received	95	90.48	p < 0.0001	0.9–1.0
*Means of accessing health care*				
Health insurance	74	70.48	p = 0.02	0.2–0.4
*Sensitizes community on TB*				
Gives talk on TB	46	43.8	p = 0.47	0.5–0.6

Only thirty-three (31.43 %) of the volunteers had registers for recording health events in the community with the majority (68.57 %; 72/105) of them without any (p < 001). Fifteen of those who had registers (45.45 %) had some health events recorded in them. Only twenty-eight of the 105 volunteers (26.67 %) had ever identified suspected TB cases in the communities. Thus, a greater proportion (77/105, 73.33 %) [p = 0.001] of the volunteers had never identified any suspected TB case in their community. Very few of the CBSV (9.52 % 10/105) [p < 0.0001] indicated that they do receive feedback from the district health management team (DHMT) on surveillance reports submitted to the district (Table 3).

### Health services delivery facilities and related TB case detection factors

**Fig. 1:**
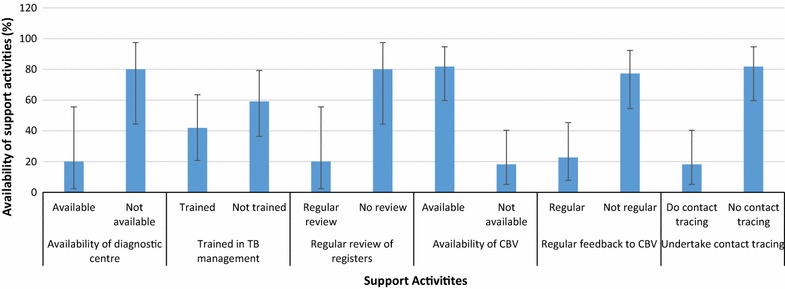
Tuberculosis support activities in the Nkwanta south district. The points plotted (*end of* *bars*) indicate the level of support activity. The *vertical lines* show the corresponding 95 % confidence intervals

The study looked at availability of diagnostic centres with capacity to carry out sputum smear examination in the district as key to improving TB case detection. There were 10 health facilities (two hospitals and eight health centres) with laboratory services [community-based health planning and services units (CHPS) do not provide laboratory services]. Only two (20.0 %) out of the 10 facilities with laboratory services were found to be carrying out sputum smear examinations. Similarly, only nine out of the 22 (40.91 %) key health care providers (physicians, physician assistants and technical officers on the TB programme) have had some training on TB management, with the majority 13 (59.1 %) of them without TB specific management training. Only two out of the 10 health facilities (20.0 %; 2/10) undertake the required monthly review of outpatient and laboratory registers to monitor progress of TB case detection and management (Fig. 1). Thus, most of the health facilities in the district were not following laid down procedures for effective and efficient TB case detection and management.

In the same way, contact tracing was found to be lacking as only four facilities had personnel who carry out contact tracing of registered TB patients into their communities. That is as many as 81.81 % (18/22) (p < 0.001) of the health facilities were not active in tracing contacts of TB patients. It was also realized that, only seven (31.81 %) out of the 22 facilities encouraged cured TB patients to help in educating and propagating the fact that TB is preventable and curable. It was however encouraging to note that most of the health facilities (81.81 %; 18/22) used the services of community health volunteers who help in various ways including disease surveillance activities. Unfortunately, however, feedback is not always given (77.27 %; 17/22) (p < 0.001) to these volunteers who help in disease surveillance activities in the district (Fig. 1).

## Discussion

The current study was conducted in the Nkwanta South district of Ghana to identify possible factors contributing to low TB case detection in the district. The work involved the review of out-patients and laboratory records for the year 2012, and primary data collection from staff of health facilities in the district, household contacts of sampled registered TB patients and community-based surveillance volunteers. Analysis of the data revealed that only 25 % of out-patients who reported persistent cough of 2 weeks and more duration had sputum examination done. Twenty-five percent of those tested were found to be positive for *Mycobacterium tuberculosis* infection. Interestingly, 9 % of the positive cases were not registered for treatment possibly as a result of lapses in the record keeping and reconciliation systems of the health facilities. The level of ‘initial defaulters’ however, was much lower than that reported from a regional hospital in an earlier study by Afutu et al. [13].

Detection of the infectious stage of tuberculosis in patients seeking treatment in our health facilities is an essential component of the TB control programme in Ghana. Based on the current record review in which 25 % of patients tested for TB were found to be positive, there is the likelihood that many more of the patients may have TB. The fact that not all patients who had cough for 2 weeks and more were tested could contribute to the low TB case detection in the district [14] and consequently the spread of the infection.

In the new policy and strategy of the National Tuberculosis Control Programme [11] of Ghana, health institutions are to carry out active case search by doing contact tracing that begins from facility records. This current study has revealed that less than one-fifth of household contacts were traced and informed of the TB status of their relatives leaving a large number uninformed. Some studies have shown that 5 % of close household contacts of TB patients have active TB infections [15]. This low level of contact tracing could also contribute to the low case detection rate.

Another factor that could be affecting the TB case detection rate in the Nkwanta South district is the lack of willingness of the community members to have their TB status checked. The fact that as high proportion of those who were informed about the TB status of their relatives did not avail themselves for testing could mean that they did not perceive themselves as high risk group for TB. On the other hand, the fear of the unknown could have affected their willingness to have themselves availed to be tested for TB infection. The fear may include social isolation by self, close relatives or friends who might get to know that one is a TB patient. Based on this assumptions it is important to build social support to reduce physical and social barriers that prevent people in high risk areas from knowing their status. Stigma and isolation of TB patients are well known social barriers that affect self-reporting for testing [16].

The availability and distribution of diagnostic centres across the district was found to be highly inadequate since only 2 out of 10 of the laboratories conduct sputum smear examination. Moreover, these two diagnostic centres were all located in the district capital, Nkwanta, which is far from some communities. Patients needing laboratory test from other locations in the district had to travel long distances to the district capital for sputum smear test and later come back for the results. Travelling long distances from difficult-to-reach communities for the results could be a serious challenge and may lead to primary defaulting if they could not come for positive results. In an earlier study in the Sisala district of the upper west region of Ghana, it was observed that travelling long distances in and out of diagnostic centres for sputum smear examination results by patients resulted in delay in giving out laboratory results [17]. The inability of patients to return for positive TB test results was found to be responsible for high defaulting and low case detection in that area.

## Conclusion

In conclusion, several factors have been identified as possible contributors to the low TB detection rate in the district. These include inadequate diagnostic centres and low level of active surveillance by health staff. Other possible factors include, weak record review systems and stigma at the community level. We believe that improving active surveillance by health staff will go a long way to improve TB case detection in the district.

## Limitations of the study

The main limitation of this study is the retrospective nature of some of the events which are subject to recall bias especially among close contacts of the TB patients. In spite of this limitation, this study gives us a good idea of possible factors contributing to low TB case detection in the Nkwanta district of Ghana.

## Additional file

10.1186/s13104-016-2136-x Data file 1: OPD record review for 2012 at Nkwanta south district hospital; Data file 2: TB clients 2012; Nkwanta south district: SSM test; Data file 3: TB clients 2012; Nkwanta south district; Data file 4: Nkwanta south district TB programme; records from TB register.

## Abbreviations

CBSV: community based surveillance volunteer
CHPS: community health planning services
DOTS: directly observed treatment short-course
TB: tuberculosis
MDG: millennium development goal
NHIS: National Health Insurance Scheme
NTP: National Tuberculosis Control Programme
NKTS: Nkwanta south
OPD: out patient department
PTB: pulmonary tuberculosis
SSA: South Saharan Africa
SSM: smear sputum microscopy

## References

[1] Borgdorff MW, Nagelkerke NJD, Dye C, Nunn P (2000). Gender and tuberculosis: a comparison of prevalence surveys with notification data to explore sex differences in case detection. Int J Tuberc Lung Dis.

[2] World Health Organization. Global tuberculosis report 2012. Geneva: WHO; 2014. http://apps.who.int/iris/bitstream/10665/91355/1/9789241564656_eng.pdf and http://www.who.int/tb/publications/global_report/gtbr13_annex_4_key_indicators.pdf.

[3] World Health Organization (2014). Global tuberculosis report 2014.

[4] World Health Organization (2002). The world health report 2002: reducing risks, promoting healthy life.

[5] Stewart GR, Robertson BD, Young DB (2003). Tuberculosis: a problem with persistence. Nat Rev Microbiol.

[6] Kik SV, Verver S, van Soolingen D, de Haas PE, Cobelens FG, Kremer K, Borgdorff MW (2008). Tuberculosis outbreaks predicted by characteristics of first patients in a DNA fingerprint cluster. Am J Respir Crit Care Med.

[7] World Health Organization (2002). The world health report 2002: reducing risks, promoting healthy life.

[8] Siddiqi K, Lambert ML, Walley J (2003). Clinical diagnosis of smear-negative pulmonary tuberculosis in low-income countries: the current evidence. Lancet Infect Dis.

[9] Amo-Adjei J, Awusabo-Asare K (2013). Reflections on tuberculosis diagnosis and treatment outcomes in Ghana. Arch Public Health.

[10] Zumla A, George A, Sharma V, Herbert N, Ilton BM (2013). WHO’s 2013 global report on tuberculosis: successes, threats, and opportunities. Lancet.

[11] Mauch V, Bonsu F, Gyapong M, Awini E, Suarez P, Marcelino B, Klinkenberg E (2013). Free tuberculosis diagnosis and treatment are not enough: patient cost evidence from three continents. Int J Tuberc Lung Dis.

[12] Nkwanta South District Health Directorate Annual Report, 2012.

[13] Afutua FK, Zachariah R, Hinderaker SG, Ntoah-Boadi H, Apori Obeng E, Adae Bonsua F, Harriese AD (2012). High initial default in patients with smear-positive pulmonary tuberculosis at a regional hospital in Accra, Ghana. Trans R Soc Trop Med Hyg.

[14] Storla DG, Yimer S, Bjune GA (2008). A systematic review of delay in the diagnosis and treatment of tuberculosis. BMC Public Health.

[15] Morrison J, Pai M, Hopewell PC (2008). Tuberculosis and latent tuberculosis infection in close contacts of people with pulmonary tuberculosis in low-income and middle-income countries: a systematic review and meta-analysis. Lancet Infect Dis.

[16] Demissie M, Getahun H, Lindtjørn B (2003). Community tuberculosis care through “TB clubs” in rural North Ethiopia. Soc Sci Med.

[17] Ahorlu CK, Bonsu F (2013). Factors affecting TB case detection and treatment in the Sissala East District Ghana. J Tuberc Res.

